# Influence of probiotic supplementation and rabbit line on growth performance, carcass yield, blood biochemistry and immune response under hot weather

**DOI:** 10.5713/ab.24.0904

**Published:** 2025-04-28

**Authors:** Magdy Abdelsalam, Moataz Fathi, Alaa El-Raffa, Ghada Abd El-latif, Osama Abou-Emera, Mohamed Abd El-Fatah, Gamal Rayan

**Affiliations:** 1 Department of Animal and Fish Production, Faculty of Agriculture, Alexandria University, Alexandria, Egypt; 2 Department of Animal and Poultry Production, College of Agriculture and Food, Qassim University, Al-Qassim, Saudi Arabia; 3 Department of Poultry Production, Faculty of Agriculture, Alexandria University, Alexandria, Egypt; 4 Department of Poultry Breeding Research, Animal Production Research Institute, Agriculture Research Centre, Giza, Egypt; 5 Department of Poultry Production, Faculty of Agriculture, Ain Shams University, Cairo, Egypt; 6 Department of Animal and Fish Production, College of Agricultural and Food Sciences, King Faisal University, Al-Ahsa, Saudi Arabia

**Keywords:** Carcass, Hot Climate, Immune Response, Probiotics, Rabbit Genotype

## Abstract

**Objective:**

An experiment was conducted to investigate the beneficial effect of probiotic supplementation via drinking water on growth performance, carcass yield, blood parameters, and immunity of two rabbit lines during the summer season.

**Methods:**

In a 6-week probiotic supplementation trial, a total of 90 healthy growing rabbits having a similar weight (590.3±16 g) representing two lines (V-Line and Alexandria) were randomly allocated into three equal groups. Three levels of probiotics (0, 6.67×10^9^ colony forming unit [CFU]/L and 13.35×10^9^ CFU/L of *Bacillus licheniformis*) were administered through drinking water as non-supplemented, low, and high levels.

**Results:**

A linearly increased body weight at 5 and 6 weeks of the experiment (p = 0.01 and p<0.01, respectively) were noticed with the probiotic supplementation at high level. The same trend was noticed for the overall body weight gain (p = 0.05). Probiotic supplementation did not affect carcass traits, except for heart and lungs, where the rabbits that received the high level recorded higher weights (p = 0.01 and p<0.01, respectively). Probiotic supplementation showed a linearly effect (p = 0.02) on the cell-mediated index after 24 h of phytohemagglutinin injection. Lower triglyceride concentration (p = 0.04) was observed in the high group compared to the other groups. Concerning the line rabbit, although the Alexandria rabbits (Alex) recorded lighter body weight, they exhibited a better gain-to-feed ratio (p = 0.05) compared to the V-line ones. Also, an increase in heart weight and spleen index (p<0.01) was found in V-Line rabbits compared to the Alex ones. Contrary, the Alex rabbits recorded a higher kidney weight (p<0.01) than that of V-Line. Overall, no difference was recorded between the rabbit lines in immune response.

**Conclusion:**

The beneficial effects of probiotic supplementation via drinking water on rabbit performance under hot weather were identified as improved growth performance, an enhanced lipid profile, and an early increase in cell-mediated immunity in both rabbit lines that received a high level of the probiotics.

## INTRODUCTION

Rabbit meat is characterized by a high protein and low-fat content, and it is considered a delicacy and a healthy food product. Because rabbit meat is low in both cholesterol and calories, it can be a great option for those looking to maintain a healthy weight or reduce their calorie intake while still enjoying a nutritious, flavorful protein source [ 1]. Due to rabbits having a short biological cycle and high prolificacy, a highly economic return from raising rabbits is gained [2]. Due to their lack of sweat glands and thick fur covering their body, rabbits are more susceptible to heat stress than most other farm animal species [3]. Conse quently, they rely on other physiological mechanisms (like panting and heat exchange through their ears) and behavioral changes to regulate their body temperature [4]. Accordingly, raising rabbits in hot climates can present significant challenges, especially when dealing with heat stress. Rabbits are naturally adapted to cooler environments and have a limited ability to regulate their body temperature, making them susceptible to heat stress [5]. There is no doubt that high environmental temperatures have been reported to induce a series of drastic changes in the biological functions of growing rabbits, leading to negative effects. Deterioration in productive performance and impairment of immunity, and consequently a higher mortality rate, were extensively reported in heat-stressed rabbits [6–8].

Replacement of antibiotics with natural feed additives in rabbit feeding is a main concern for organic livestock farming. Probiotics are live microorganisms, typically bacteria or yeast that are beneficial to health when supplemented with animal feed in adequate amounts. These beneficial bacteria, such as *Lactobacillus, Bifidobacterium*, and *Saccharomyces*, can support and improve digestive efficiency through suppressing the growth of harmful pathogens, such as *Escherichia coli* and *Clostridium* species, in rabbits. Probiotics have been hypothesized to be effective as natural growth promoters in animal feeding. Using probiotics as a natural feed additive in rabbit feeding strongly enhanced productivity, immunocompetence, and carcass yield under high environmental temperatures [9–11]. Growth performance, feed efficiency, and health status were improved in rabbits fed a diet supplemented with probiotics [9,12 ]. The probiotics act to activate beneficial microbes in the gut, improve the immune system, demonstrate positive antimicrobial properties, and improve the intestinal histomorphometry, feed digestibility, and nutrient absorption in rabbits [13,14].

With respect to genotypic contribution, rabbit breeds do differ in their productive performance under heat stress. Genotypes that have originated in warmer climatic regions or have been selectively bred for heat tolerance tend to perform better under hot conditions [15]. These breeds may have natural adaptations, such as lighter coats or greater heat dissipation capabilities. In recent years, there has been increasing interest in crossbreeding the Egyptian rabbit with other breeds to enhance heat tolerance and improve productive performance under heat stress. A developed paternal rabbit line, named Alexandria, was originated and established in Alexandria University, Egypt. A crossing between V-line and black Egyptian rabbits was adopted and applied to produce this developed line to be superior for daily weight gain at 63 days of age [16]. Local hybrid rabbits exhibited a better response in terms of most immunological parameters when compared with exotic rabbits under hot ambient temperatures [17]. The present study aimed to evaluate the beneficial effects of commercial probiotic supplementation (*Bacillus licheniformis*) via water on the growth performance, carcass yield, immune response and biochemical blood analysis in two rabbit lines raised in hot environmental conditions.

## MATERIALS AND METHODS

### Study site and ethical approval

The current experiment was conducted during the hot summer season (July to August) of 2024 at the poultry research center belonging to Alexandria University, Alexandria, Egypt. All procedures relating to live experimental rabbits used in the study were performed and approved in accordance with the guidelines of the Experimental Animal Care Committee Ethics of the Alexandria University (Protocol # Alex.Agri. 092401101). All efforts were made to ensure a minimum of animal suffering.

### Experimental design, animals and management

Two different rabbit lines was used in the present experiment. V-line is an exotic Spanish rabbit line imported from Valencia Polytechnic University, Spain, that were selected for giving more weaned kittens per litter. The Alex rabbit line is a developed line produced from crossing between the V-line and Egyptian native breed to be more acclimatized for hot environmental conditions. A total of 90 weaned rabbits representing two lines (45 V-line and 45 Alex line) aged four weeks of age were utilized in a 6-week experiment. The rabbits of each line were randomly distributed into 3 levels of probiotic supplementation in a 2×3 factorial arrangement comprising 6 sub-groups (15 rabbits each). The initial body weight was not different (590.3±16 g) for all sub-groups. The rabbits of each sub-treatment were collectively divided into 5 wire-fenced cages (5 replicates, 3 rabbits each). The dimensions of the cage are 50 cm×40 cm×38 cm. The cages were installed in one-level cage construction located in an open-sided building. Each cage was supplied with a hopper feeder and two drinking nipples. The experiment started after a one-week adaptation period, during which the rabbits were acclimatized to the new environment and to the presence of the staff. The animals were raised under similar environmental and managerial conditions. The average of maximum and minimum ambient temperatures throughout the whole experimental period was 32.2±0.2°C and 25.9±0.2°C, respectively. The average of relative humidity inside the house was 71.5±2.2%. All animals were given *ad libitum* access to a commercial pelleted ration containing 18% crude protein, 12% crude fiber, and 2,650 kcal/kg metabolizable energy. The feed and water were offered to rabbits *ad libitum* throughout the whole experimental period. For probiotic supplementation, a substance containing 6.67×10^9^ colony forming units (CFU)/g of *Bacillus licheniformis* (GalliPro-Tect-WS; Chr. Hansen, Milwaukee, WI, USA) was used in two levels. For low and high concentration levels of probiotics, 1 g and 2 g were dissolved in each liter of drinking water, respectively. The actual *Bacillus licheniformis* concentration was 6.67×10^9^ CFU/L and 13.35×10^9^ CFU/L for low and high levels, respectively. The rabbit group that drank the fresh water without supplementation represents a non-supplemented group. To ensure the effective dissolving of the probiotics in drinking water and delivery in a fresh manner to the rabbits, the mixture was prepared and placed daily in a water tank for each group.

### Body weight and feed intake

The growth performance, including weekly body weight and overall feed intake, was determined. The total body weight gain from the initial weight over six weeks was calculated. Gain-to-feed ratio (G:F) was calculated based on overall body weight gain and feed intake for each experimental unit (3 rabbits).

### Slaughter and carcass evaluation

At the end of the experiment, 10 rabbits were randomly assigned from each group (60 rabbits in total) for carcass characteristics. They were fasted for 12 h with free access to clean drinking water and slaughtered. Upon bleeding, the rabbits were dissected. After skinning, the carcass was eviscerated, and all organs and offal were removed. Hot carcass, skin, head, liver, heart, kidney, and lungs were excised and weighed. Cecum with content was weighed, and the length was measured using a measuring tape. Also, lymphoid organs (spleen and thymus) were removed and trimmed. The carcass was divided into three cuts: fore part, mid part, and hind part.

### Blood collection and biochemical analysis

Blood samples were collected post-mortem from each rabbit into heparinized tubes for determination of biochemical analysis. The blood samples were centrifuged (805×g for 15 minutes at 4°C), and the harvested plasma was stored at −20°C until further analysis. Total protein, albumin, cholesterol, triglycerides, high-density lipoprotein (HDL), and low-density lipoprotein (LDL) were determined in the plasma using commercial kits (Biomerieux, Marcy-l’Étoile, France). The globulin was calculated as the difference between the total protein and albumin.

### Cell-mediated immunity assay

During the fifth week of the experimental period (10 weeks of age), a total of 72 rabbits were randomly assigned (12 animals/subgroup) for cell-mediated immune response. The in vivo response induced by injecting a mitogen was evaluated by injecting phytohemagglutinin (PHA-P) into the left ear. Each rabbit was injected intradermally with 100 μg PHA-P (Sigma Chemical, St. Louis, MO, USA) in 0.1 mL of sterile saline. Upon injection, the needle site was marked with a permanent black marker to detect the site of further measuring. The resultant swelling response in the ear was measured with a constant tension dial micrometer (Ames, Waltham, MA, USA) before injection and at 24, 48, and 72 h post PHA-P injection. Ear swelling was expressed as the difference between the thickness of the ear before and after injection. Cell-mediated index was calculated as a relative swelling response to the first measure.

### Statistical analysis

Data were subjected to a two-way analysis of variance using JMP software version 13.0 with line and probiotic level as fixed effects [18]. The statistical model is described as follows:


$${\text{Y}}_{\text{ijk}}=\mu +{\text{P}}_{\text{i}}+{\text{L}}_{\text{j}}+{\left(\text{PL}\right)}_{\text{ij}}+{\text{e}}_{\text{ijk}}$$

where:

Y_ijk_ = the observation taken on the k^th^ individual,
μ = overall mean,
P_i_ = the fixed effect of the j^th^ probiotic supplementation level,
L_j_ = the fixed effect of the i^th^ line,
(PL)_ij_ = interaction between line and probiotic supplementation level,
e_ijk_ = random error assumed to be independent normally distributed with mean = 0 and variance = σ^2^.

All results were presented as mean, and the variability in the data was expressed as pooled standard error of the mean. The significance of difference among the groups was assessed using Tukey’s test. Differences between means were considered significant at p≤0.05. Orthogonal polynomial contrast test for linear and quadratic effects was applied to describe the shape of the response to increasing concentrations of probiotic supplementation and to determine the best model fit. The responses in optimal parameters to the probiotic supplementation level can be modeled using the following quadratic equation:


$$\text{Y}=\text{a}+{\text{b}}_{1}{\text{X}}_{1}+{\text{b}}_{2}{\text{X}}_{2}+\text{e}$$

where: Y = optimal response, a = intercept, bs = coefficients of the quadratic equation, Xs = probiotic levels, and e = error.

## RESULTS

### Body weight and feed intake

Weekly body weight and overall growth performance of the studied rabbit lines (Alex and V-line) fed different level of probiotic supplementations are listed in Table 1. No differences were observed due to probiotic supplementation or line during the first 4 weeks of the experimental period. A linear difference (p = 0.01) was observed in body weight at the end of the fifth week among the probiotic treatment groups. The high probiotic level group (1,351 g) recorded a higher body weight (p<0.05) compared to the non-supplemented one (1,288 g), whereas the rabbits given low probiotic level (1,316 g) had an intermediate weight that was not different from the other two groups. Also, at the end of the study (11 weeks of age), it could be noticed that the inclusion rate of probiotics had a linearly increased (p = 0.01) in body weight. Regarding the rabbit line effect, V-line rabbits recorded a higher (p<0.05) body weight at the fifth week of the experiment compared to the Alex rabbit line. This increase continued until the end of the experiment (p = 0.09). Overall, no differences in feed intake and G:F were detected among probiotic supplemented groups. The rabbits received high level of probiotics recorded an increase of body weight gain (p = 0.05) in linear manner compared with that of non-supplemented group. The rabbit received the low level of probiotics was intermediate. Concerning rabbit line effect, the results indicated that Alex rabbits consumed a lower (p<0.01) feed intake (2,906 g) with improved (p = 0.05) G:F (0.307) compared with V-line rabbits (3,175 g and 0.280, respectively). There was no interaction between probiotic level and rabbit line for all growth performance traits.

### Carcass characteristics and organ weights

**Figure 1:**
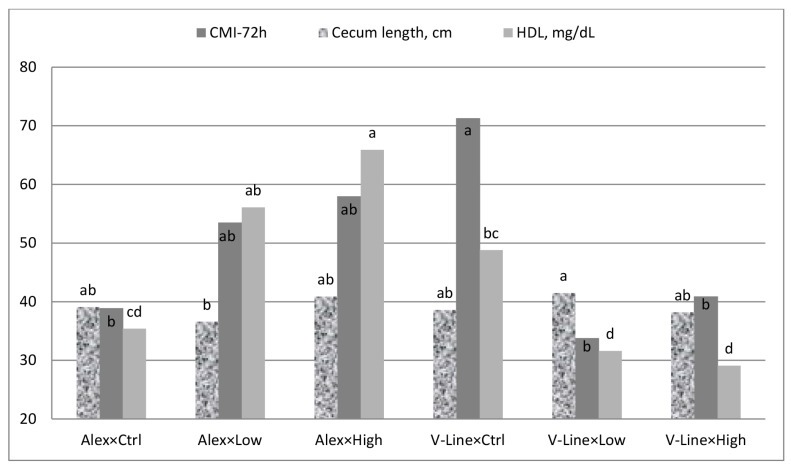
Effect of interaction between probiotic supplementation level (ctrl, low and high) and rabbit line (Alex and V-Line) on cell-mediated index-72 h, cecum length and HDL concentration. ^a–d^ The bars with no common superscripts within each parameter are different (p<0.05). HDL, high-density lipoprotein.

Table 2 shows the carcass characteristics and organ weights of growing rabbits as influenced by probiotic supplementation and rabbit line. Generally, most carcass traits did not differ either for probiotic supplementation level or for rabbit line. There was a difference in heart weight among the probiotic levels (linear manner, p = 0.01), with the high probiotic group (6.7 g) having a higher heart weight than the non-supplemented group (5.1 g). The low probiotic group (5.8 g) had intermediate heart weight values. Entirely, almost a 4% increase in carcass weight of supplemented groups compared to non-supplemented rabbits. Rabbit line also had a positive effect on heart weight (p<0.05), with V-Line rabbits (7.4 g) showing larger hearts than Alex rabbits (4.2 g). In terms of kidney weight, the Alex rabbits recorded a higher weight (p<0.05) compared to the V-Line rabbits (9.8 g and 7.1 g, respectively). Although the main two effects were not different for cecum length, there was an interaction between them (p = 0.03). Within the low probiotic level, the Alex rabbits recorded a shorter cecum (36.6 cm) compared with V-Line ones (41.5 cm; Figure 1).

### Blood biochemical analysis

The results indicate that both probiotic supplementation levels and rabbit line can influence various blood parameters, but their effects vary across the parameters measured (Table 3). The total protein level did not show an effect due to probiotic supplementation. However, a rabbit line effect was observed, with the Alex line exhibiting higher total protein level (5.28 g/dL) compared to V-Line (4.91 g/dL, p = 0.04). Albumin and globulin levels did not show differences either with respect to probiotic supplementation or line effects. Cholesterol level was not affected by probiotic supplementation or rabbit line. Triglyceride level showed a linearly response (p = 0.04) to probiotic supplementation, with the high probiotic group showing a lower concentration (56.5 mg/dL) compared to the non-supplemented group (63.6 mg/dL) and low probiotic groups (64.1 mg/dL). Furthermore, a rabbit line effect (p<0.01) was observed, with the Alex line having a higher level of triglycerides (74.7 mg/dL) compared to the V-Line rabbits (49.2 mg/dL). There was no interaction between probiotic level and rabbit line. In terms of the two types of lipoproteins, LDL did not show differences for probiotic addition level and rabbit line. Moreover, the interaction between them did not show an effect. With respect to HDL level, an effect for probiotic supplementation (p = 0.01; in linear manner) and rabbit line (p<0.01) was detected. Probiotic supplementation at the high level resulted in a higher (p<0.05) HDL (53.6 mg/dL) compared to the low probiotic and non-supplemented groups (43.3 mg/dL and 42.8 mg/dL, respectively). Concerning the line effect, the Alex line recorded a higher (p<0.01) HDL value (53.1 mg/dL) compared to the V-Line one (38.7 mg/dL). Moreover, an interaction (p<0.01) between probiotic level and rabbit line was found for HDL concentration. The Alex rabbits recorded the highest concentration (65.9 mg/dL), while the V-Line recorded the lowest figure (29.1 mg/dL) within the high level of probiotics (Figure 1).

### Cell-mediated immunity response

The cellular-mediated response of growing rabbits given different levels of probiotics and lymphoid organ index are listed in Table 4. Probiotic supplementation showed a linear effect on cell-mediated index 24 h post-injection. A notable increase in the response (p = 0.02) was recorded for rabbits receiving the high level of probiotics (92.4) compared to the non-supplemented and low-level groups (70.8 and 73.2, respectively). At 48 and 72 h post-injection, neither probiotic supplementation nor rabbit line exhibited a difference. There was no interaction between probiotic level and rabbit line except for the last determination after 72 h (p<0.01). The V-Line rabbits that did not receive probiotics recorded the better cell-mediated index (71.3) compared to the other sub-groups (Figure 1). In terms of lymphoid organs, there was no effect of probiotic level on the spleen index. However, a difference (p<0.01) between rabbit lines was found. The V-Line exhibited a higher (p<0.01) spleen index (1.2) compared to the Alex line (0.93). A linear difference (p = 0.04) in thymus index was detected among rabbit groups given different probiotic levels. The rabbits that received a high level of probiotics had a larger (p = 0.04) thymus index (4.2) compared to the non-supplemented and low-level groups (3.4 and 3.7, respectively). However, rabbit line and the interaction did not affect the thymus index.

## DISCUSSION

### Growth performance

During the first 4 weeks of the 6-week experimental period, neither the supplemented groups nor the rabbit lines showed any differences in body weight. These results suggest that probiotic supplementation continues to influence body weight in the later stages of growth, with high levels being most effective. There was no interaction between probiotic level and rabbit line for all growth performance, suggesting that the effects were consistent across lines. The groups given probiotics showed greater body weight than the non-supplemented group, supporting the potential role of probiotics in enhancing growth performance during the later stage of the growing period (10 to 11 weeks of age). These findings are consistent with previous studies that have reported improved growth performance with probiotics in various livestock species [ 19]. They also found that the advantage of supplementing feed with probiotics for growing rabbits was more pronounced in summer conditions. Similar results were reported by [20], who found an increase in body weight and growth performance in rabbits fed a ration supplemented with a mineral mixture and probiotic may be due to increased digestion and absorption through the intestine. It has been shown that feeding rabbits with probiotics may have a growth-promoting activity by competing with harmful gut flora and stimulating the immune system [9]. Conversely, Copeland et al [21] did not find any difference in body weight gain between non-supplemented and probiotic-fed groups in a long-term neonatal rabbit model. Feed intake determined throughout the experimental period did not show a difference among probiotic groups. Specifically, the V-Line exhibited a greater feed intake than that of the Alex line. G:F ratio describes the efficiency with which an animal converts feed into body weight gain. A higher G:F ratio indicates that the animal is gaining more weight for each unit of feed consumed, meaning more efficient in converting feed into body gain. Though the probiotic level did not affect G:F ratio, a trend towards improved G:F ratio in the groups that received probiotics was observed. The lack of changes in feed intake and G:F ratio due to probiotics might indicate that the effects of probiotics on growth are independent of feed consumption and that they might work through other mechanisms such as improved gut health, nutrient absorption, or immune modulation. The analysis indicates that the line has a statistically effect, with the Alex line having a better G:F ratio compared to the V-Line, suggesting that Alex rabbits may be genetically more efficient in converting feed into body weight. This result agrees with the findings of [9], who reported that the native rabbit breeds exceeded the foreign ones, maybe due to a negative effect of high ambient temperatures on growth performance. Higher growth performance coupled with slightly improved G:F ratio in rabbits receiving probiotics might be accounted for increased digestibility of all the nutrients [22].

### Carcass characteristics and organ weights

The results suggest that probiotics had a limited impact on organ weights and carcass characteristics. However, probiotic supplementation did influence heart weight and lung weight, both of which were greater in the high probiotic group. This could indicate that probiotics may promote the development of specific organs, particularly those involved in metabolism and immune function, such as the heart and lungs. However, for most traits (e.g., liver, kidneys, and cecum weight), probiotics did not lead to changes, suggesting that probiotics may not affect these organs under the given conditions. Bhatt et al [22 ] and Saini et al [23] suggested that dietary supplementation of probiotics improved body weight gain, but carcass characteristics were not influenced in rabbit. Likewise, Ahmed et al [24] reported that probiotic supplementation did not affect the weight of the internal organs. Furthermore, genetic line differences play a significant role in certain traits, particularly heart and kidney organs. The line difference was more noticeable for kidney weight, with the Alex line showing greater weight, while no line effects were observed for other traits like live body weight or lung weight. These findings suggest that the rabbit line factor may play a larger role in certain organ developments, like kidneys, than probiotic supplementation. It is worthy to note that the V-line breed recorded lower carcass weight and dressing percentage compared to the other genetic groups, including the native rabbits [9]. Similarly, Wang et al [25] found that breed had an effect on liver and kidney percentages. Cecum weight was not influenced by either the probiotic treatment or the rabbit line, but there was an interaction between the two factors was found in cecum length. In accordance with this finding, Rotolo et al [26] reported that caecum weight was not affected by treatment in weaning rabbits upon dietary inclusion of a probiotic (live *Saccharomyces cerevisiae boulardii*). However, further research would be needed to understand the underlying mechanisms of the interaction between probiotics and genotype under different environmental conditions. A slight increase in carcass weight in rabbits that received probiotics was observed compared to non-supplemented rabbits. This may be due to the superiority of their live body weight at slaughter.

### Blood biochemical parameters

Data regarding blood biochemical analysis revealed that the administration of probiotics did not affect the blood concentration of total protein, albumin, globulin, or cholesterol. These results are partially in agreement with [ 27], who reported that there is no effect on plasma total proteins after dietary *Lactobacillus acidophilus* supplementation of growing rabbits. Contrary to our results, an increase in the concentration of total protein and albumin was reported in New Zealand rabbits following a supplementation with *Saccharomyces cerevisiae* over an 8-week experimental period [28]. Concerning rabbit line, the Alex rabbits recorded a higher total protein level compared to the V-line rabbits. This increment could be attributed to a better absorption and utilization of nutrients by the intestine [29]. The current results revealed that the probiotic supplementation and the rabbit line did not influence cholesterol concentration. Triglyceride level showed a probi otic effect, with lowering level found in rabbits receiving high probiotic supplementation. Moreover, an increase in HDL was recorded in high-level rabbits compared with low-level or non-supplemented groups. Several studies have explored the role of probiotics in lipid metabolism, showing that they can impact serum cholesterol levels, particularly lowering LDL and triglycerides while sometimes increasing HDL [30]. This is consistent with the findings of the current study, where probiotic levels appeared to influence triglyceride and HDL levels but not LDL. Sjofjan et al [31] reported that the administration of probiotics in rabbit feed affects the mechanisms of lowering lipids in blood transportation followed by a reduction in triglycerides. Lowering levels of triglycerides are correlated with the decrease of remnant lipoprotein and the redistribution of lipids from plasma to target organs, in this case the liver [9]. On the other hand, Abdulrahim et al [32] reported that the addition of *Lactobacillus acidophilus* reduces the cholesterol in the blood by deconjugating bile salts in the intestine, thereby preventing them from acting as precursors in cholesterol synthesis. Regarding the rabbit line effect, a previous study conducted by Fathi et al [9] suggested that the genetic group of rabbits interacted with dietary probiotic supplementation for some blood parameters under hot environmental conditions. In this context, the results indicated that probiotics might have a positive impact on total protein and lipid profile, especially in terms of HDL synthesis in specific lines, such as Alex rabbits. Additionally, the effects of probiotics seem to vary based on the rabbit genetic line, where the interaction was found. Accordingly, the blood chemistry of rabbit breed or genotype may be varied when applying probiotics in animal feeding.

### Immune response and lymphoid organs

The results suggest that probiotics can stimulate cellular immune response, especially after a short term of the test (24 h post-injection), but the effects vary depending on the time frame and probiotic dose. Likewise, an impact on specific lymphoid organs could be detected resulting from probiotic supplementation levels and rabbit lines. The higher probiotic supplementation enhances the cell-mediated immune response at 24 hours, which is a critical component of the immune system’s defense against pathogens. While the differences among supplemented groups disappeared thereafter. The rabbit group that received a high probiotic level recorded a higher thymus index compared to the other groups. Several studies have stated that using probiotics as feed additives markedly improved the immune response and disease resistance in rabbits [9,33, 34]. Probiotics improve the immune response by stimulating the mucosal immune system, maintaining the intestinal barrier by modulating the gut microbiota, and producing microbial inhibitory compounds [35]. Additionally, using probiotics in diet alleviated thermal stress-induced negative effects and enhanced the immune response and intestinal health of growing rabbits reared during summer conditions [36]. The spleen index, which is a measure of spleen size and often correlates with immune activity, was found to be higher in V-line than that of Alex one. This suggests that the genetic background may influence organ size independently of probiotic supplementation, indicating a more robust immune response. Dietary supplementation with *Bacillus subtilis* increased immune organs’ indices, intestinal homeostasis, immune response, and disease resistance [37]. However, the difference between the present results and some previous studies may be attributed to using different probiotic strains and various types of rabbit genotypes raised under varied environmental conditions.

## CONCLUSION

Supplementation of drinking water with *Bacillus licheniformis* markedly improves the growth performance across both rabbit lines, particularly during the later age of a 6-week experimental period. Also, a high level of probiotics supported lipid profile by lowering triglycerides and increasing HDL levels and improved cellular immune response after a short term of PHA-P injection. The V-line rabbits (foreign rabbits) recorded a heavier body weight and higher feed intake compared to the Alex line (improved Egyptian rabbits), while the latter recorded a lower feed intake and improved G:F. Moreover, higher levels of blood total protein and HDL were bserved in Alex rabbits. Probiotic supplementation was not the only influencing factor in the current study. The interaction between the two factors must also be taken into consideration. Probiotic supplementation as a growth-promoting agent via drinking water in rabbits appears promising under hot weather irrespective of genetic line. However, probiotic effectiveness should be evaluated in conjunction with other factors such as rabbit genotype and the type of probiotic administration as well as the prevailing temperature.

## Figures and Tables

**Table 1:** Weekly body weight (g) and overall growth performance of the studied rabbit lines (Alex and V-line) fed different level of probiotic supplementations

Parameter	Probiotic level (P)			Line (L)		SEM	p-value			
	
	Ctrl	Low	High	Alex	V-Line		P		L	P×L

							Linear	Quad		
Initial body weight	587.9	588.6	594.4	579.3	601.3	15.7	0.871	0.943	0.499	0.984
1^st^ week body weight	752.0	767.9	744.8	749.0	761.3	17.1	0.855	0.597	0.748	0.720
2^nd^ week body weight	845.2	861.8	889.3	861.7	868.8	18.8	0.349	0.894	0.856	0.865
3^rd^ week body weight	1,008	1,022	1,036	1,007	1,038	19.7	0.564	0.954	0.447	0.793
4^th^ week body weight	1,171	1,215	1,220	1,187	1,217	20.0	0.344	0.626	0.452	0.611
5^th^ week body weight	1,288^b^	1,316^ab^	1,351^a^	1,286^b^	1,353^a^	11.4	0.013	0.776	<0.001	0.086
6^th^ week body weight	1,458^b^	1,488^ab^	1,512^a^	1,473	1,499	18.6	<0.001	0.956	0.093	0.072
Body weight gain (g)^1)^	862.4^b^	898.3^ab^	918.1^a^	892.3	891.8	17.1	0.054	0.255	0.972	0.640
FI (g)	3,012.4	3,025.5	3,084.5	2,906.3^b^	3,175.3^a^	52.8	0.902	0.498	<0.001	0.135
G:F ratio	0.286	0.296	0.297	0.307^a^	0.280^b^	0.006	0.803	0.886	0.048	0.184

1) Body weight gain, weight gain from the initial weight over six weeks.

a,b The means with no common superscripts within each factor are different (p<0.05).

Alex, Alexandria line; SEM, standard error of the mean; FI, overall feed intake; G:F, overall gain-to-feed ratio.

**Table 2:** Effect of supplemented probiotic level and rabbit line on carcass characteristics and the organ weights of growing rabbits

Trait	Probiotic level (P)			Line (L)		SEM	p-value			
	
	Ctrl	Low	High	Alex	V-Line		P		L	P×L

							Linear	Quad		
Live body weight (g)	1,482	1,500	1,536	1,512	1,499	15.6	0.164	0.843	0.749	0.607
Skin (g)	154.9	165.9	169.2	169.6	157.4	4.02	0.184	0.619	0.155	0.888
Head (g)	93.1	99.0	97.4	96.4	96.7	1.19	0.143	0.121	0.880	0.358
Heart (g)	5.1^b^	5.8^ab^	6.7^a^	4.2^b^	7.4^a^	0.37	0.013	0.704	<0.001	0.128
Kidneys (g)	8.8	8.6	7.7	9.8^a^	7.1^b^	0.35	0.092	0.472	<0.001	0.456
Liver (g)	38.9	39.1	39.3	38.8	39.3	0.75	0.851	0.960	0.693	0.374
Lungs (g)	7.7^b^	8.1^ab^	8.9^a^	8.1	8.2	0.20	<0.001	0.655	0.632	0.411
Cecum length (cm)	38.8	39.2	39.7	38.8	39.5	0.59	0.659	0.921	0.634	0.025
Cecum weight (g)	110.6	104.2	98.2	100.9	107.7	3.52	0.177	0.963	0.402	0.136
Front (g)	270.0	287.6	285.5	277.7	284.2	4.34	0.128	0.254	0.417	0.410
Mid (g)	162.5	165.2	162.1	159.6	166.8	3.35	0.968	0.685	0.298	0.919
Hind (g)	314.7	326.9	326.6	320.0	325.3	4.96	0.312	0.527	0.554	0.628
Carcass weight (g)	747.2	779.8	774.2	757.4	776.3	11.40	0.304	0.410	0.384	0.637

a,b The means with no common superscripts within each factor are different (p<0.05).

Alex, Alexandria line; SEM, standard error of the mean.

**Table 3:** Effect of supplemented probiotic level and line on blood biochemical analysis in growing rabbits

Parameter	Probiotic level (P)			Line (L)		SEM	p-value			
	
	Ctrl	Low	High	Alex	V-Line		P		L	P×L

							Linear	Quad		
Total protein (g/dL)	5.23	5.00	4.98	5.28^a^	4.91^b^	0.10	0.161	0.574	0.037	0.158
Albumin (g/dL)	3.32	3.41	3.22	3.36	3.28	0.05	0.407	0.143	0.373	0.322
Globulin (g/dL)	1.97	1.59	1.76	1.92	1.63	0.09	0.344	0.177	0.111	0.546
Cholesterol (mg/dL)	116.5	122.9	127.7	127.3	118.1	4.4	0.506	0.263	0.344	0.061
Triglycerides (mg/dL)	63.6^a^	64.1^a^	56.5^b^	74.7^a^	49.2^b^	2.4	0.040	0.251	<0.001	0.358
LDL (mg/dL)	49.9	49.6	42.7	45.7	49.3	1.8	0.181	0.379	0.384	0.823
HDL (mg/dL)	42.8^b^	43.3^b^	53.6^a^	53.1^a^	38.7^b^	2.4	0.009	0.408	<0.001	<0.001

a,b The means with no common superscripts within each factor are different (p<0.05).

Alex, Alexandria line; SEM, standard error of the mean; LDL, low-density lipoprotein; HDL, high-density lipoprotein.

**Table 4:** Effect of supplemented probiotic level and rabbit line on cell mediated index and lymphoid organs of growing rabbits

Parameter	Probiotic level (P)			Line (L)		SEM	p-value			
	
	Ctrl	Low	High	Alex	V-Line		P		L	P×L

							Linear	Quad		
CMI^1)^-24 h	70.8^b^	73.2^b^	92.4^a^	76.9	80.2	3.64	0.022	0.271	0.594	0.070
CMI-48 h	84.6	63.1	70.3	76.5	68.4	4.72	0.144	0.161	0.363	0.078
CMI-72 h	54.4	43.7	50.7	50.0	49.2	3.95	0.549	0.286	0.847	<0.001
Spleen index^2)^	1.09	1.1	1.1	0.93^b^	1.2^a^	0.05	0.966	0.984	<0.001	0.353
Thymus index^3)^	3.4^b^	3.7^b^	4.2^a^	3.8	3.7	0.17	0.044	0.725	0.933	0.912

1) $\text{Cell-mediated index }(\text{CMI})=\frac{\text{swelling induced at a tested time }-\text{ear thickness at 0 time}}{\text{ear thickness at 0 time}}\times 100$.

2) $\text{Spleen index}=\frac{\text{spleen weight (g)}}{\text{live body weight (g)}}\times 100$.

3) $\text{Thymus index}=\frac{\text{thymus weight (g)}}{\text{live body weight (g)}}\times 100$.

a,b The means with no common superscripts within each factor are different (p<0.05).

SEM, standard error of the mean.
